# Exploration of Blood Lipoprotein and Lipid Fraction Profiles in Healthy Subjects through Integrated Univariate, Multivariate, and Network Analysis Reveals Association of Lipase Activity and Cholesterol Esterification with Sex and Age

**DOI:** 10.3390/metabo11050326

**Published:** 2021-05-18

**Authors:** Yasmijn Balder, Alessia Vignoli, Leonardo Tenori, Claudio Luchinat, Edoardo Saccenti

**Affiliations:** 1 Laboratory of Systems and Synthetic Biology, Wageningen University & Research, Stippeneng 4, 6708 WE Wageningen, The Netherlands; yasmijn.balder@wur.nl; 2 Magnetic Resonance Center (CERM) and Department of Chemistry “Ugo Schiff”, University of Florence, Via Luigi Sacconi 6, 50019 Sesto Fiorentino, Italy; vignoli@cerm.unifi.it (A.V.); tenori@cerm.unifi.it (L.T.); luchinat@cerm.unifi.it (C.L.); 3 Consorzio Interuniversitario Risonanze Magnetiche MetalloProteine (CIRMMP), Via Luigi Sacconi 6, 50019 Sesto Fiorentino, Italy

**Keywords:** analysis of biological networks, lipid metabolism, lipidomics, metabolomics, nuclear magnetic resonance

## Abstract

In this study, we investigated blood lipoprotein and lipid fraction profiles, quantified using nuclear magnetic resonance, in a cohort of 844 healthy blood donors, integrating standard univariate and multivariate analysis with predictive modeling and network analysis. We observed a strong association of lipoprotein and lipid main fraction profiles with sex and age. Our results suggest an age-dependent remodulation of lipase lipoprotein activity in men and a change in the mechanisms controlling the ratio between esterified and non-esterified cholesterol in both men and women.

## 1. Introduction

Lipids are the most abundant biological molecules in human plasma [1]. This group of small molecular weight molecules shows large structural and functional variations: they are fundamental building blocks of the cell wall and are key components of the cell membrane and other cellular compartments, including the nuclear membrane, the endoplasmic reticulum, and the Golgi apparatus, as well as trafficking vesicles such as endosomes and lysosomes [2]. 

Mammalian cells express tens of thousands of different lipid species and use hundreds of proteins to synthesize, metabolize, and transport them: the diversity of lipids is of the same order of magnitude as that of proteins, but until recent years they were not studied as much as proteins [2].

Lipids are transported in the blood by proteins; lipoproteins exist in different densities: chylomicrons, very low-density lipoprotein (VLDL), low-density lipoprotein (LDL), intermediate-density lipoprotein (IDL), and high-density lipoprotein (HDL). These lipoproteins determine where the lipid is transported to, which contributes to the wide functional variability of the lipidome. 

It is widely recognized that variations in lipoprotein profiles and metabolism are associated with metabolic diseases such as diabetes mellitus and cardiovascular diseases, and can thus be used to monitor and assess the risk of such diseases. For instance, elevated levels of LDL cholesterol [1] and triglycerides [3,4] increase the risk of cardiovascular diseases, while high levels of HDL cholesterol are correlated with a low risk of cardiovascular diseases [1,3,5]. Moreover, alterations of lipoprotein profiles have been associated with different types of cancer [6,7,8] and autoimmune diseases [9,10,11].

Variations in the blood lipid profiles are associated not only with particular pathophysiological statuses but also with sex and age: women tend to have higher levels of triglyceride VLDL than men [12], whereas men have higher total triglycerides and cholesterol levels [13], and the overall concentration of VLDL in men increases with age, while it decreases in women [5]. 

Many more sex- and age- lipoprotein associations are being discovered [5,14,15,16]: in the era of precision medicine, understanding how sex and age shape the lipidome can lead to better diagnosis and treatment of conditions that occur more frequently in one of the two sexes, present sex-specific symptoms and outcomes, or are characteristic of a specific age group [17].

In this study, we investigate sex- and age-specific differences in the plasma lipidome of 844 young and middle-aged healthy blood donors of both sexes who were analyzed for their lipoprotein blood profiles via nuclear magnetic resonance (NMR) spectroscopy [18,19].

We integrated standard univariate analysis, multivariate exploratory analysis, and predictive modeling with systems biology tools to explore the relationships among lipoprotein fractions using association networks and differential network analysis. Since lipoprotein concentrations change in an orchestrated fashion, the patterns of associations between lipoprotein fractions can be considered, to some extent, related to the underlying structure of the biological networks [20]. Differences in lipoprotein associations which are sex- and age-related can indeed point to affected molecular mechanisms since changes can be more significant than levels alone [21,22], as shown in applications to health, sex, and age phenotyping [17,23], cardiovascular risk [24,25,26], and bacterial infections [27,28].

Here, we report the findings of this integrated analysis describing how sex and age affect both the concentration and the correlation patterns of lipoprotein profiles in healthy subjects, and we suggest that lipids may be used as an early biomarker to monitor healthy aging.

## 2. Results

**Figure 1:**
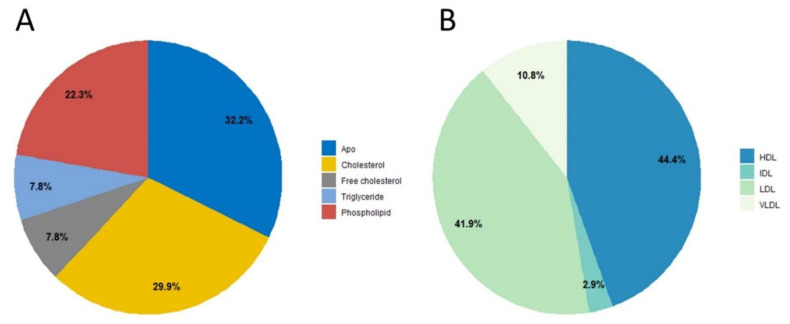
Relative concentration (%) of lipoproteins and lipids (**A**) and lipid main fractions (**B**) in the study cohort comprising 844 heathy blood donors (661 men + 183 women). Very low-density lipoprotein (VLDL), low-density lipoprotein (LDL), intermediate-density lipoprotein (IDL), and high-density lipoprotein (HDL).

An overview of the lipid fractions and lipoprotein distribution in the overall study group (men and women) is shown in Figure 1. Age characteristics are given in Table 1.

### 2.1. Univariate Analysis: Lipoprotein and Lipid Fraction Concentrations Differ between Sexes and Age Groups

The concentrations of lipoprotein main fractions are markedly different between men and women as shown in Table 2, where we observed: 16 fractions have a different concentration in men with respect to women (7 out of 16 are elevated). When comparing young and old men, we observed 13 lipoprotein and lipid fractions to have a lower concentration in the young men. In women we found only 5 lipid fractions to have a lower concentration in young women. ROC analysis was also performed to assess the discriminatory capability of the lipoprotein main fractions and to define the best. Results are given in Table 3, Table 4 and Table 5.

Apo-A1 and Apo-A2 (HDL), cholesterol (HDL), free cholesterol (HDL), and phospholipids (HDL) are specific lipoprotein fractions which discriminate between men and women (AUC = 0.689, 0.656 and 0.715, respectively). Triglycerides (LDL) are unique in differentiating young and old men (AUC = 0.720), while free cholesterol (LDL) and phospholipids (LDL) are unique for the discrimination between young and old women (AUC = 0.820 and 0.782). 

### 2.2. Multivariate Analysis and Predictive Modeling Indicate the Existence of Sex- and Age-Specific Lipoprotein and Lipid Fraction Profiles

**Figure 2:**
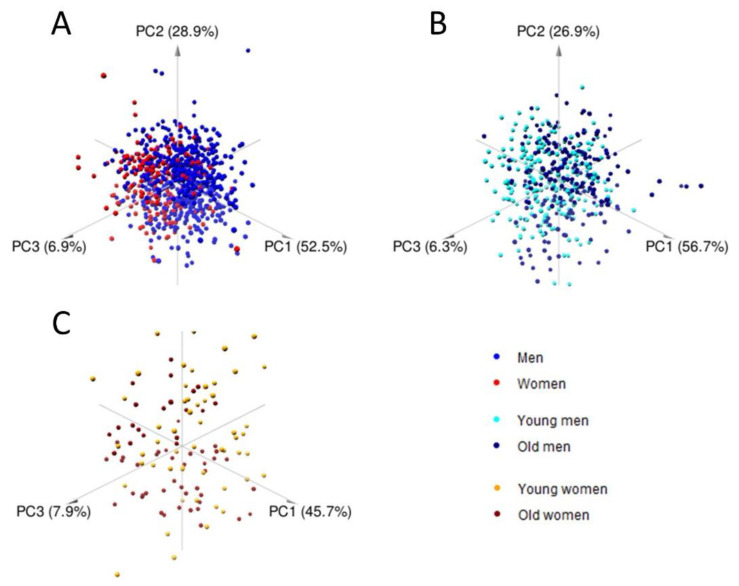
Principal Component Analysis (PCA) score plot of lipoprotein main fraction profiles for men and women (**A**), young and old men (**B**), and young and old women (**C**).

Principal component analysis (PCA) was performed on the 21 lipoprotein main fractions for the complete dataset of men and women, on young and old men, and on young and old women separately; scatter plots for the first three principal components for the three PCA models are shown in Figure 2A–C. There is a slight separation between the sex and age groups, indicating either that differences in lipoprotein profiles are subtle or that separation happens in a high-dimensional space and hence cannot be visualized.

We applied a Tracy–Widom test to verify the underlying dimensionality of the data, and we found that 19, 18, and 19 principal components (for the M-W, YM-OM, and YW-OW data, respectively) are statistically significant at the 0.001 level, which indicates that they summarize signal information and not noise, also showing the necessity of considering high-order components to fully describe the data. 

We built Random Forest classification models to investigate the predictive capability of lipoprotein main profiles to distinguish between men and women and between the age groups. Model quality measures are reported in Table 6. All models are statistically significant at the α = 0.01 level, with relatively high AUC, indicating the presence of sex- and age-specific blood lipoprotein signatures. 

**Figure 3:**
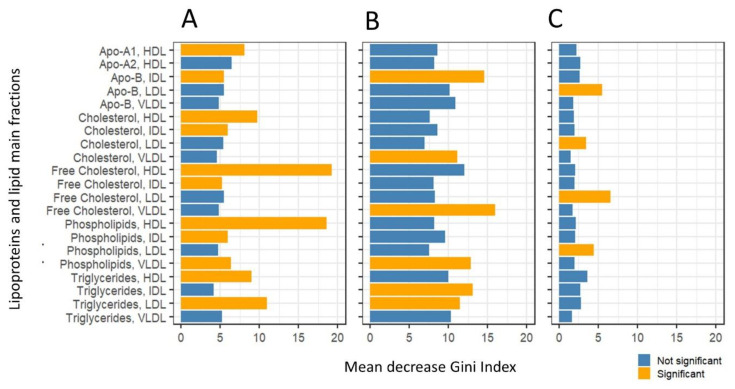
Importance of lipoprotein main fractions in the sex and age group classification models built with Random Forest. (**A**) Men vs women, (**B**) young men vs old men, (**C**) young women vs old women. Variable importance is expressed as Mean Decrease Gini index. Variables with *p*-value < 0.05 are highlighted in orange. *p*-values are not corrected (see Results Section 2.2).

Variable importance was obtained for each model as Mean Decrease Gini index [29], and statistical significance was assessed by means of a permutation testing procedure. Results are shown in Figure 3. We observed specific signatures in the discriminant models: for instance, separation of age groups (young vs old) in women is attributable to LDL fractions, while discrimination between male age groups is dominated by VLDL fractions. We observed here that while several variables contribute significantly (*p*-value < 0.05) to the model(s), if correction for multiple testing is applied, none remain significant (see Figure 3 caption for more details). This conflicts with the relatively strong discriminant models obtained (see Table 6): this loss of power is probably attributable to the non-independence of tests and/or how the permutation testing is implemented in the rfPermute package which was used to calculate the statistical significance of the Mean Decrease Gini index.

### 2.3. Network Inference and Analysis

There is a wealth of information contained in the relationships among plasma and blood metabolites [28,30,31], which are better captured using correlation measures as an index of association [32,33]. Lipoprotein main fraction association networks were built using the PCLRC algorithm and a Gaussian Graphical Model approach to estimate the pairwise partial correlations among the concentrations of the lipoprotein fractions.

Networks were built separately for men’s and women’s data and for the corresponding age groups. Results are shown in Figure 3, for a total of six networks, each comprising 21 nodes. 

Partial correlations measure degree of association between two variables when removing the effect of other controlling variables and were used in place of standard correlation to avoid the risk of introducing indirect correlations in the network modeling. Partial correlation networks allow the modeling of unique interaction among the variables (lipoproteins) and can be indicative of potential causal pathways [34]: a non-zero partial correlation would be expected if (*i*) A causes B, (*ii*) B causes A (*iii*) there is a reciprocal relationship between A and B or (*iv*) both A and B cause a third variable in the network [34,35,36]. Moreover, if variables covaries because of variables that are not present in the network, it is expected that all these variables will be connected in the network, forming a cluster [36]. 

#### Exploratory Analysis of Lipoprotein and Lipid Fractions Highlights Subtle Remodulation of Correlation Patterns

**Figure 4:**
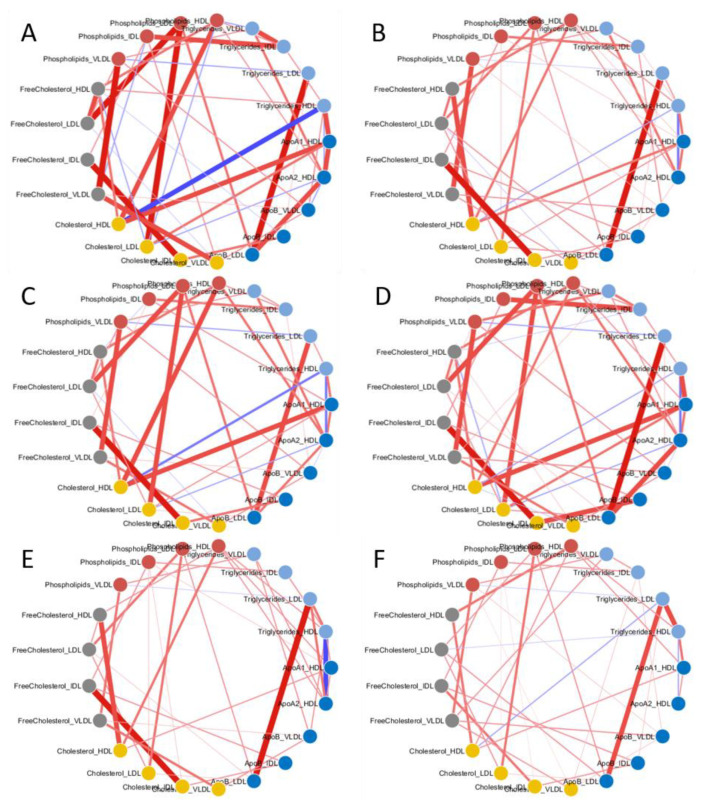
Association networks of lipoprotein and lipid main fractions (nodes) for (**A**) women, (**B**) men, (**C**) young men, (**D**) old men, (**E**) young women, and (**F**) old women. Associations (edges) are quantified by partial correlation estimated with a Gaussian Graphical Model [37] in combination with the PCLRC probabilistic algorithm [25]. Positive partial correlations between nodes are colored in red; negative partial correlations between nodes are colored in blue. The edge weights are proportional to the edge weight. Nodes are color-coded according to the 5 lipid groups considered: Apo (dark blue), cholesterol (yellow), free cholesterol (grey), phospholipids (red), and triglycerides (light blue).

The overall structure of the networks is similar across the the study groups (men/women, young/old) and differences most depends on variation of the strenght of the associations: for instance, the correlation between cholesterol and triglycerides (HDL) is stronger in the association network for men (Figure 4B) than in that for women (Figure 4A). 

**Figure 5:**
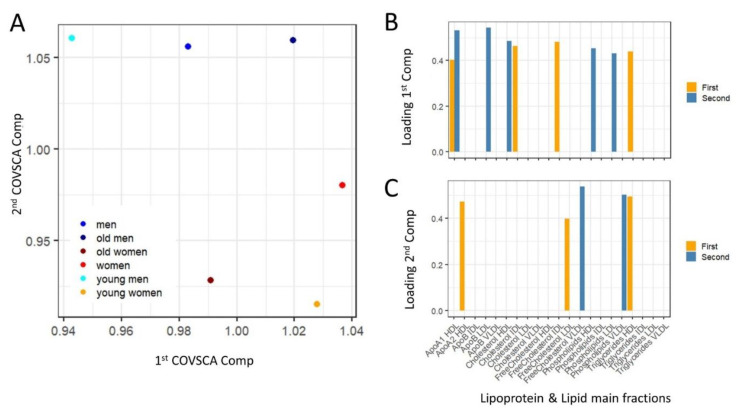
Covariance Simultaneous Component Analysis of the lipoprotein and lipid fractions association networks for all male subjects (men), all female subjects (women) and young/old men and young/old women. (**A**) COVSCA score plot: each dot is a low-dimensional representation of lipid association network. (**B**,**C**) Loadings associated with the two COVSCA components: since the COVSCA model is fitted with two rank-2 prototype matrices there are two sets of loadings for each component (see Equation (6)). The loadings describe the relative importance of each lipoprotein and lipid fraction in describing the different correlation structure observed in the network specific to each sex and age group. Loadings were filtered on the basis of *z*-score: only loadings with *z*-score > 1 have been retained.

To explore comprehensively the patterns of variation of the asociation strenght, Covariance Simultanous Component Analysis was applied to analyze simultaneously the six networks and to individuate which lipoprotein fractions show different correlation patterns across the networks. The score plot of the COVSCA analysis is shown in Figure 5A and can be interpreted in a PCA-like fashion: points close in the COVSCA space share similar characteristics. Since every point represents a network, this indicates network similarities.

The association networks separate according to sex along the second COVSCA dimension (note the in COVSCA, in contrast with PCA, the order of the dimensions is arbitrary: dimensions can be swapped without changing the model), indicating the existence of correlation patterns among lipoprotein fractions that are sex-specific. Networks separate by age groups along the first COVSCA dimension, showing age-dependent correlation patterns. 

It is interesting to note that male age groups separate perfectly along the second dimension, with the network built using all male subjects fallowing in-between the networks for the young and old groups, and this is somehow expected. For women-specific networks, the picture is slightly more complicated: the age groups separate along the first dimension as for males, but the network obtained with all female samples partially separate along the second component, suggesting a more complicate remodulation of correlation patterns.

The COVSCA model was fitted with two rank 2 prototype matrices (as the best compromise between model complexity and goodness of fit (38.2%), since COVSCA is an exploratory approach) which results in two set of loadings for each dimension that are shown in Figure 5B (first dimension) and Figure 5C (second dimension): as in PCA the COVSCA loadings describe the importance of each variable/lipoprotein to the model. 

Two (quasi) orthogonal sets of loadings were obtained after pruning loading with *z*-score < 1, with only the loading associated with LDL triglycerides common to the two dimensions, indicates the involvement of different sets of lipoprotein fractions in defining the correlation structure of the sex- and age-specific association networks.

The first dimension (along which networks separate by age group) is characterized mainly by cholesterol, phospholipids and apo-1 and apo-2 fractions, while the second dimension is characterized by the unique contribution of VLDL (free cholesterol and phospholipids). 

### 2.4. Differential Network Analysis Indicates Relevant Topological Differences in Lipoprotein and Lipid Fractions Specific to Sex and Age Group 

For each of the 21 lipoprotein main fractions we calculated 15 different topological measures, in addition to node degree/connectivity (Equation (A1)) with the aim of summarizing the node characteristics within each network; the topological measures are listed in Appendix A. These measures can be used to characterized the importance or the relevance of a node within a network [38]: for instance, the clustering coefficient (Equation (A4)), provides a measure of the level of interconnectivity, while centrality (Equations (A2) and (A3)) centrality identify the most important nodes within a graph. Taken together, high centrality and low clustering coefficient define a hub node, i.e., a highly connected node, in this case a lipid which is correlated with many other lipids which are thus may be key players in the network [39].

**Figure 6:**
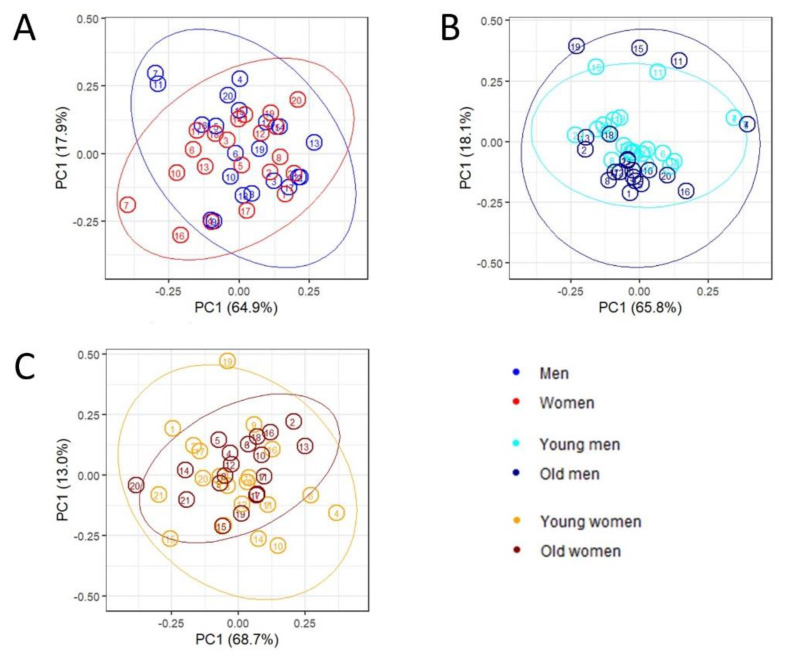
PCA of topological measures with ellipses for 95% confidence interval multivariate normal distribution. (**A**) between men (blue) and women (red). (**B**) between young (cyan) and old men (dark blue). (**C**) between young (orange) and old women (dark red). The lipoprotein main fractions are numbered as follow: 1: Apo-A1 HDL, 2: Apo-A2 HDL, 3: Apo-B VLDL, 4: Apo-B IDL, 5: Apo-B LDL, 6: Cholesterol VLDL, 7: Cholesterol IDL, 8: Cholesterol LDL, 9: Cholesterol HDL, 10: Free cholesterol VLDL, 11: Free cholesterol IDL, 12: Free cholesterol LDL, 13: Free cholesterol HDL, 14: Phospholipids VLDL, 15: Phospholipids IDL, 16: Phospholipids LDL, 17: Phospholipids HDL, 18: Triglycerides VLDL, 19: Triglycerides IDL, 20: Triglycerides LDL, 21: Triglycerides HDL.

To visualize and investigate how and to which extent node characteristics change between men’s and women’s networks and between age groups we first apply PCA on the matrix containing topological measures (columns) for each lipid (rows), in such a way each node is defined by a 15-dimensional vector. PCA score plots are shown in Figure 6. The scatter plots show that the overall node characteristics are different between different conditions, since points corresponding to the same node do not usually follow close to each other. However, a larger spread can be observed in the case of the men vs women network, indicating larger topological differences. 

We quantified topological differences at node level taking two approaches: (*i*) for each node we calculated the cosine distance in the 15-dimensional space defined by the 15 topological measure between two conditions (men vs women networks and young vs old networks) and (*ii*) we focused on node degree as defined in Equation (A5) in Appendix A.

Using approach (*i*) we selected the lipid fractions-lipoproteins/nodes with overall larger topological differences by taking the *z*-score (z). This resulted in a small set of lipoproteins and lipid fractions:

Free cholesterol HDL (*z* > 2) and HDL Apo2, ApoB VLDL, triglycerides (*z* > 1) are the lipoprotein fractions with the largest topological differences when comparing men’s and women’s networks. The comparison of age groups resulted in IDL free cholesterol (*z* > 2) and IDL triglycerides (*z* > 1) (young vs old men) and HDL ApoA2 (*z* > 1) and HDL triglycerides (*z* > 2) (young vs old women).

To complete the analysis, we took approach (*ii*) to single out lipoprotein fractions whose node degree (connectivity) was different in the networks specific to different study groups. 

Highly connected nodes, the so-called “hubs”, play a special role in biological network since there is ample evidence hub-nodes are key elements in characterizing network behavior, as observed in the case of gene co-expression and regulatory networks [40,41], metabolic networks, protein–protein interaction networks [42,43,44], and cell–cell interaction networks [45], and it has been shown that in yeast, for instance, proteins that are highly connected are essential for survival [40,42]. 

**Figure 7:**
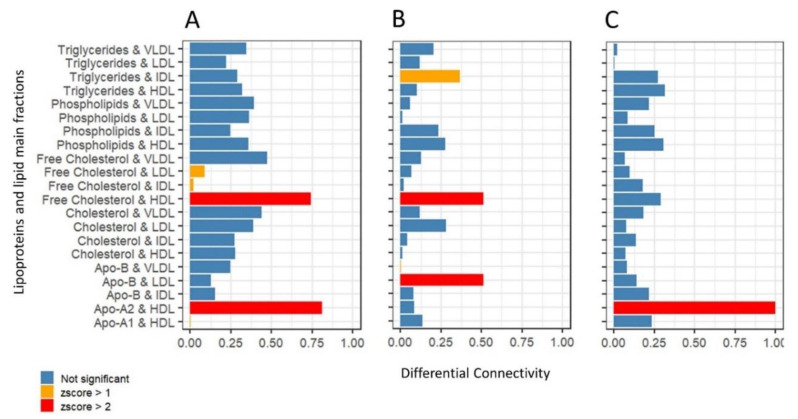
Differential Connectivity Analysis: comparison of node (lipoprotein) connectivity in the main lipoprotein fraction association networks of (**A**) men vs women; (**B**) young men vs old men; (**C**) young women vs old women. Differential connectivity is calculated using Equation (A5). Bars are color-coded by *z*-score value (Equation (5)).

Relevant differentially connected lipoproteins were selected again using *z*-scoring (see Figure 7). HDL Apo-A2 (*z* > 2) is differentially connected when comparing men- and women-specific networks, while in the comparison of age group we found HDL triglycerides and LDL ApoB IDL free cholesterol (*z* > 2) to be differentially connected in the men and HDL Apo2 (*z* > 2) to be differentially connected in women.

## 3. Discussion

In this study, we combined standard univariate analysis and ROC analysis with multivariate exploratory (PCA and COVSCA), predictive (Random Forest) modeling, and network analysis to investigate the association of blood lipoprotein main fractions with age and sex. A summary of the relevant findings from the different analyses is given in Table 7.

### 3.1. Considerations Regarding Confounding Factors

Before discussing the results of the present analysis, we shall comment that lipidic profiles may be influenced by factors other than sex and age such as diet, lifestyle, (patho)physiological conditions, genetics, and interactions thereof [46,47,48]. Dietary intake is an important factor influencing circulating lipid profiles: for example, high carbohydrate intake might lead to an increase in blood triglycerides [49] while dietary fat differently impacts the ratios of total to HDL cholesterol and LDL cholesterol [48], as well as adherence to particular dietary regimes such as vegan or vegetarian [50]. We do not possess information about participants’ dietary and lifestyle habits other than those that are not allowed as per regulations about blood donation (see Materials and Methods, Section 4.1 for an overview), so it is not possible to control for those factors. However, the cohort is extremely homogenous regarding baseline characteristics, so although an influence of lifestyle habits cannot be ruled out, it can be realistically considered to be minimal.

### 3.2. Considerations Regarding Group Size

The study groups are different in size, with more men than women, and this reflects what is observed in the blood donor demographics: women are often under-represented among blood donors, and this difference is particularly strong in Italy [51]. Within sex, we compared age groups of the same size, thus excluding bias due to different sample sizes. When results are compared across sexes, it should be considered that analysis has been performed on groups of different sample sizes: however, in all cases, sample size is large enough to allow detection of univariate effects larger than 0.5 (Cohen *d* [52]) with 80% power at the α = 0.01 level; observed effects are much larger as shown in Table 2. Predictive modeling was performed (men vs women) using resampling to create equal-sized groups. Networks were inferred on data size allowing estimation of correlation > 0.3 with 80% power at α = 0.01 level. Taken together, this indicates the robustness of our analysis. 

### 3.3. Sex Affects Lipoproteins and Lipid Fraction Profiles in Healthy Subjects

Differences in the lipd profiles of men and women are mostly attributable to HDL, regarding either differences in concentration or its associations with other lipidic fractions.

HDL particles contribute the removal of excess free cholesterol from the peripheral tissues and deliver it into the liver for excretion, in a process known as ‘cholesterol reverse transport’ [53,54]. HLD removes LDL, the main catabolic product of VLDL, in vascular space and delivers cholesterol into the tissues [55]; LDL particles can pass via the intima layer of vascular beds and be taken up by macrophages to make foam cells, thus exerting an atherogenic effect [56], and esterification of cholesterols plays a key role in this process.

We observed significantly higher levels of HDL in women than in men, and lower levels of LDL in women than in men (although the difference is not statistically significant). Elevated levels of HDL in women have been previously reported in different subpopulations [57,58,59,60,61,62,63], and lipid control by endogenous estrogens in women has been proposed, which also explains the observation that pre-menopausal women have fewer cardiovascular complications than men [12]. 

Lipids are mobilized between tissues mainly as fatty acids released by adipose tissue or as lipoprotein produced by the liver and gut (chylomicrons and very low-density lipoprotein (VLDL) for triglyceride (TG), and low-density lipoprotein (LDL) and high-density lipoprotein (HDL) for cholesterol) [12]. Metabolism and catabolism of hepatic fatty acid, triglyceride, and cholesterol is regulated by endogenous estrogens and androgens. It is understood that estrogens mediate their effects through three receptors, estrogen receptor alpha (ERα), estrogen receptor beta (ERβ), and G-protein coupled estrogen receptor (GPER), but little is known about the role of androgens [12]. However, it has been suggested that effects of progestogens and androgens mimic only in part the differences in plasma lipids between men and women and that the factors mediating the sex-specific regulation of plasma lipid kinetics and concentrations are still to be elucidated [64].

Analysis of lipid association networks shows a negative association between HDL cholesterol and HDL triglycerides (Figure 4A,B) which is physiologically consistent [65]. The ratio between HDL triglycerides and cholesterol (TG/HDL-C ratio) can be viewed as an index of insulin resistance based on comparisons of the ratio to measures of insulin resistance [66,67], and there is evidence for the association of high TG and low HDL-C with resistance to insulin-stimulated glucose uptake by peripheral tissues independently of body habitus and physical fitness, and that insulin resistance has an effect on plasma insulin, TG, and HDL concentrations [68]. 

Comparison of the lipid association networks shows that the TG and HDL cholesterol association is stronger in women than in men (despite the smaller number of women in the study): reduction or modification in the correlation between the concentration profiles may indicate remodulation or rewiring of metabolic or biochemical processes involving these molecules [33]. Reduced correlation of HDL triglycerides and cholesterol in men may reflect the well-known sexual dimorphism of insulin resistance and sensitivity [69,70,71]: women show higher insulin sensitivity (hence lower resistance) than men, and this has also been confirmed by studies using animal models [69,72,73].

We also observed remodulation of the ratios between HDL free cholesterol and HDL cholesterol and LD free cholesterol and LDL cholesterol, but the remodulations are more pronounced when comparing the age groups and will be discussed later.

### 3.4. Consideration Regarding Age Groups

Conventionally, “elderly” has been defined as a chronological age of 65 years old or older [74], and the World Health Organization uses this convenience thresholding [75,76]. 

Aging is a continuous process, and different functions are differentially affected by aging: muscle mass decreases by approximately 3–8% per decade after the age of 30 [77]; the thymus shrinks from birth at a rate of approximately 3% per year until middle age, and at a rate of 1% per year thereafter [78]; women fertility peaks around the late 20s and then starts declining [79,80]. 

This study cohort is relatively young: 43 years for women and 40 years for men, as reported in Table 1, and we have set the thresholds (see Table 1) based on the observed age distributions in men and women [17], thus investigating the extremes of the distribution, purposively excluding the middle age groups, to avoid combinatorial increase in possible comparisons and to maximize the power of the analysis to investigate what can be considered early effects of aging in a healthy population.

### 3.5. Age Affects Lipoproteins and Lipid Fraction Profiles in Healthy Subjects 

The analysis of lipidome profiles of the young and old groups indicates that aging (although this a relatively young study group) may affect men and women in a different way; however, the landscape is much more nuanced. 

Overall, the lipid fractions responsible for the differences in the lipidomic profiles of men’s and women’s age groups are vastly the same and are highlighted by several analyses (see Table 7). We observed age effects on IDL triglycerides only in men, while the LDL phospholipid fraction is affected only in women. 

LDL, which is cholesterol-rich, is formed in the bloodstream through the catabolism of VLDL at the surface of blood vessels, and the production of a cholesterol-rich lipoprotein from a triglyceride-rich lipoprotein occurs by selective removal of triglyceride from VLDL [81]. IDL can be cleared after uptake by the liver or can be processed to become LDL, and inefficient clearance of IDL tends to lead to increased LDL production [81]. We observed significantly increased concentration of all IDL fractions in old men with respect to young men, in fact suggesting reduced IDL clearance; however, we observed only a significant increase in LDL triglycerides.

Increased IDL concentrations may result from decreased clearance due to reduced lipoprotein lipase (LPL, an enzyme that catalyzes the hydrolysis of triglycerides in triglyceride-rich lipoproteins [82]) activity on the vascular endothelium, which, in turn, may result from either decreased synthesis or inadequate binding of this enzyme by HSPG (heparan sulphate proteoglycans, a class of glycoproteins, containing one or more covalently attached heparan sulphate chains, a type of glycosaminoglycan [83]). The lipoprotein lipase activity of adipose tissue has been found to decrease with age in male rat and mouse models [84,85], and this reduction seems to be less prominent in female animals [84,85]. Interestingly, network analysis reveals (Figure 4C,D and Figure 7B) remodulation of the correlation patterns of IDL triglycerides and in particular a marked decrease in the correlation between IDL triglycerides and ILD phospholipids, which also suggests remodulation of lipoprotein lipase activity. Taken together, our results indicate possible age-dependent remodulation of lipase lipoprotein activity in men but not in women. Several studies show that lipoprotein lipase activity is more stable in women than in men: for instance, muscle and adipose tissue LPL activity was found to be increased significantly in men but not in women after physical exercise [86] and to be higher in women than in men [87]. Another study, while reporting no difference in LPL activity between sexes, reported that LPL mRNA was 160% higher in women than in men [88], which suggests enhanced regulation in women that could explain the higher resilience of LPL in women with respect to external stimuli and aging.

We observed a significantly increased concentration of LDL (specifically cholesterol, free cholesterol, phospholipids, and apo-B) in older women with respect to younger ones. The increase in LDL levels is consistent with the observation that in women the transition towards menopause results in a loss of the estrogen cardioprotective effect. Although the study group is relatively young, the median age of the “old women” group is 55 years: we cannot ascertain the menopausal status of the subjects (information missing in the clinical records), but we should consider that in Italy the average menopausal age can be placed around 49–51 years [89,90,91]; thus, the “old women” study group can probably be considered to be in a peri-menopausal status.

Many mechanisms have been proposed by which endogenous estrogens may protect against cardiovascular disease, including antioxidant effects [92], antiplatelet effects [93] and, of course, remodulation of plasma lipid profiles [94,95]. During menopause, endogenous estradiol (E2) levels decrease, accompanied by an increase in LDL cholesterol with plasma levels that can exceed those of age-matched men [95], a trend that we also observed in this study (Table 2). 

As previously mentioned, differential network analysis suggests an age-dependent remodulation of the association between HDL free cholesterol and HDL cholesterol and LD free cholesterol and LDL cholesterol. This remodulation is present in both men’s and women’s networks (Figure 4C–F).

Cholesterol is present as unesterified (free) and esterified portions in the body fluids, and while free cholesterol is biologically active and has cytotoxic effects, the cholesteryl ester (CE) is a protective form for storage in the cells and transporting in plasma [53,96]. The free cholesterol is shielded by first being converted to acyl ester, which then binds to proteins by taking part in lipoprotein structure [53]. LDL and HDL possess atherogenic and antiatherogenic properties, respectively, and it has been suggested that the atherogenicity may be determined by the ratio between unesterified (free) and esterified cholesterol [53]. Unesterified cholesterol is mobilized from peripheral tissues and other lipoproteins to HDL, with the help of transporters of ABC-A1, ABC-G1, and receptors of SR-B1 [97]. In HDL, free cholesterol is esterified rapidly by LCAT (lecithin-cholesterol acyltransferase) and cofactor of apoA I [98]. Esterification causes a concentration gradient and draws in cholesterol from tissues or other lipoproteins. Finally, cholesteryl esters are transferred to lighter fractions by CETP (cholesteryl ester transfer proteins) to deliver to the liver for excretion [53].

We observed a reduction in the strength of the association between HDL free cholesterol and HLD cholesterol in both old men and women, which suggests that mechanisms controlling the ratio between the esterified and non-esterified cholesterol may undergo a remodulation with age. Although altered activity of LCAT has been observed in several diseases such as cancer [99] and diabetes [100], the relationship between variations in plasma LCAT activity and subclinical atherosclerosis is unclear [101]. Increased activity of LCAT has been observed in metabolic syndrome and suggested to be a marker of subclinical atherosclerosis, indicating that elevated LCAT is not necessarily beneficial for cardioprotection [101], and other studies on Type 2 diabetes subjects indicate that increased LCAT activity may contribute to impaired or reduced antioxidative functionality of HDL [102]. Lecithin-cholesterol acyltransferase mass was found to be correlated with both enzyme activity and the molar cholesterol esterification rate, and its mass to be positively correlated with age [103]: this leads us to speculate that what is reflected in the modification of lipid association networks may be increased LCAT activity leading to a reshaping of the ratios between esterified and non-esterified HDL cholesterol.

## 4. Materials and Methods

### 4.1. Study Population 

The study population includes 844 healthy volunteers, of which are 183 women and 661 men, with a median age of 43 ± 12 yrs and 40 ± 11 yrs, respectively. The participants in this study were selected from the Tuscany section of the Italian Association of Blood Donors (AVIS) in the Transfusion Service of the Pistoia Hospital. 

Plasma samples were obtained according to the Italian guidelines for blood donations (Annex III of the Decree of the Italian Ministry of Health of 2 November 2015 on “Provisions relating to the quality and safety requirements of blood and blood components”), restricting donors to age 18−60 years, body weight > 50 kg, systolic blood pressure 110−148 mmHg, diastolic blood pressure 60−100 mmHg, hemoglobin > 13.5 g/dL for men and >12.5 g/dL for women; absence of (manifested) infectious diseases, absence of chronic diseases, no current menstruation, no consumption of medicines within 1 week before donation (bd) or according to the active substance and the pharmacokinetics of the prescribed drug and the disease being treated, no common diseases (such as flu, cold, bronchitis) within 2 weeks bd, no surgery within 3 months bd, no endoscopic exams within 4 months bd, no pregnancy within 12 months bd, no abortion within 4 months bd, no travel to tropical countries within 6 months bd, and, in particular, no sport activity within 24 h bd. Further details can be found in previous publications [17,25,26].

### 4.2. Study Data

We made use of publicly available data [104]. NMR data used to quantify lipoproteins and lipid main fractions are available in the Metabolights database [105] with accession number MTBLS147 [25]. Full details on sample collection and NMR experiments are given below for the reader’s convenience.

### 4.3. Sample Collection and Handling

All samples were collected under a fasting condition. Ethylenediaminetetraacetic acid (EDTA) plasma samples were collected and handled according to Standard Operating Procedures as described in [106] and stored at −80 °C until NMR analysis at the time of the original study [104]. 

### 4.4. NMR Sample Preparation

Frozen plasma samples were thawed at room temperature and shaken before use [106]. A total of 300 μL of a sodium phosphate buffer (10.05 g Na_2_HPO_4_·7H_2_O; 0.2 g NaN_3_; 0.4 g sodium trimethylsilyl [2,2,3,3-^2^H_4_] propionate (TMSP) in 500 mL of H_2_O with 20% (*v*/*v*) ^2^H_2_O; pH 7.4) was added to 300 μL of each plasma sample, and the mixture was homogenized by vortexing for 30 s. A total of 450 μL of this mixture was transferred to a 4.25 mm NMR tube (Bruker BioSpin srl, Rheinstetten, Germany) for analysis.

### 4.5. NMR Analysis and Lipoprotein Quantification

One-dimensional ^1^H NMR spectra for all plasma samples were acquired, at the time of the original study [104], using a Bruker 600 MHz spectrometer (Bruker BioSpin) operating at 600.13 MHz proton Larmor frequency and equipped with a 5 mm CPTCI ^1^H-^13^C-^31^P and ^2^H-decoupling cryoprobe including a z-axis gradient coil, automatic tuning-matching (ATM), and an automatic sample changer. A BTO 2000 thermocouple served for temperature stabilization within an uncertainty of ~0.1 K at the sample. Before measurement, samples were kept for at least 3 min inside the NMR probehead for temperature equilibration at 310 K. One-dimensional water-suppressed Nuclear Overhauser Effect SpectroscopY pulse sequence (NOESY 1Dpresat) was used to obtain NMR spectra in which signals of both low-molecular and high-molecular weight components are present; 64 Free induction decays (FIDs) were collected into 98,304 data points over a spectral width of 18,028 Hz, with an acquisition time of 2.7 s, a relaxation delay of 4 s, and a mixing time of 0.01 s. FIDs were multiplied by an exponential function equivalent to a 1.0 Hz line-broadening factor before applying Fourier transformation. Transformed spectra were automatically corrected for phase and baseline distortions and calibrated (glucose anomeric doublet at 5.24 ppm) using TopSpin 3.2 (Bruker Biospin srl).

Lipoprotein quantification was performed using the Bruker IVDr Lipoprotein Subclass Analysis platform™ (Bruker Biospin). This approach utilizes a PLS regression model to perform lipoprotein subclass analysis on ^1^H NMR NOESY spectra [107,108]. The main VLDL, IDL, LDL and HDL classes, six VLDL subclasses VLDL-1 to VLDL-6, six LDL sub-classes LDL-1 to LDL-6, four HDL-subclasses HDL-1 to HDL-4 were quantified. Only the lipoprotein main fractions (Apo-A1 HDL, Apo-A2 HDL, Apo-B VLDL, Apo-B IDL, Apo-B LDL, Cholesterol VLDL, Cholesterol IDL, Cholesterol LDL, Cholesterol HDL, Free cholesterol VLDL, Free cholesterol IDL, Free cholesterol LDL, Free cholesterol HDL, Phospholipids VLDL, Phospholipids IDL, Phospholipids LDL, Phospholipids HDL, Triglycerides VLDL, Triglycerides IDL, Triglycerides LDL, Triglycerides HDL) were taken into account in the present analysis.

The dataset is available at the NIH Common Fund’s National Metabolomics Data Repository (NMDR) website, the Metabolomics Workbench, www.metabolomicsworkbench.org where it has been assigned as Study ID ST001785.

### 4.6. Definition of Age Groups

Study subjects were divided into young (Y) and old (O) using the same approach used in [17], taking as boundaries the lower 33% and upper 67% percentiles of the age distribution of men and women, separately. Since the two distributions are different, age boundaries for the Y and O groups are also different for men and women. Percentiles and group size are shown in Table 1. 

### 4.7. Statistical Analysis

#### 4.7.1. Data Pre-Processing

Data were adjusted for heteroscedasticity by taking the square root of concentrations [109,110]. 

#### 4.7.2. Univariate Analysis 

The Mann–Whitney–Wilcoxon rank-sum test [111] was used to compare and assess statistical significance of differences in lipid concentrations between the groups of interest: men vs women, young men vs old men, and young women vs old women. Bonferroni multiple testing correction [112] was applied to reduce the risk of false positives. An adjusted *p*-value < 0.01 was deemed significant.

#### 4.7.3. ROC Analysis

Analysis of receiver operating characteristic (ROC) curves [113] was performed to assess the accuracy of the concentration of lipoproteins and lipid main fractions in discriminating between subject groups (W-M, YM-OM, YW-OW). ROC analysis allows testing of accuracy over the entire range of protein concentration, and it does not require a predetermined cut-off point to distinguish between discrimination and non-discriminating proteins; in addition, ROC analysis is not dependent on the group size. The area under the ROC (AUC) was obtained for each lipoprotein and lipid main fraction independently when comparing the study groups, with the associated 95% confidence interval, the corresponding accuracy, specificity and sensitivity and the estimated best concentration threshold; 95% CI were computed with 2000 stratified bootstrap replicates.

The statistical significance of the AUC was obtained as the *p*-value of the corresponding Mann–Whitney–Wilcoxon test [111], since the *U* test statistic is equivalent to the area under the receiver operating characteristic curve (AUC = *U*/*n*_1_*n*_2_ where *n*_1_ and *n*_2_ are the size of the two groups) since the same Null Hypothesis is tested [114,115].

#### 4.7.4. Multivariate Analysis

##### Exploratory Analysis

Principal Component Analysis (PCA) [116,117,118] was applied to explore data patterns. Data were scaled to unit variance before analysis [110]. 

##### Dimensionality Assessment

Data dimensionality assessment, which is the determination of the number of significant principal components needed to describe information but not noise in the data, was performed testing the eigenvalues of the covariance data matrix using a testing procedure based on the Tracy–Widom distribution [119,120,121]. Briefly, the test compares the eigenvalues with the eigenvalue distribution that is expected under the null hypothesis of all variables being uncorrelated. A significance threshold of α = 0.001 was used. The test was applied on the (covariance matrix calculated from the) full men’s and women’s datasets and on the young/old men and young/old women, separately.

##### Predictive Modeling

The Random Forest (RF) algorithm [122,123] was employed for pairwise classification of the lipid profiles of men and women, young men and old men, and young women and old women. Predictive models were built using a repeated cross-validation: the data were divided into a training set, with which the model was build based on the lipoprotein main fractions, and a testing set, with which the model was validated. 

Data unbalance was taken into account for the model comparing men’s and women’s lipid profiles by using a stratified data sampling, sampling datasets of equal size from both men’s and women’s datasets: the sample size is equal to that of 85% of the smallest of the two groups; 100 resampling iterations were performed to take into account the (re)sampling variability.

Accuracy, sensitivity, specificity, and the area under the receiver operating characteristic were calculated according to the standard definitions [124]. Average values and 95% confidence interval (CI) are calculated over the 100 resampling. 

Significance of the model was determined using a permutation-test: Random Forest predictive models were built after class labels were randomly permuted *k* = 1000 times to build a null distribution *D^perm^* for each model quality from which the corresponding *p*-values were calculated as (for AUC):

(1) $$p-value{|}_{\mathrm{AUC}}=\frac{1+\#\left(D_{AUC}^{perm}\geq {\mathrm{AUC}}_{0}\right)}{K}$$

AUC_0_ indicates the AUC value for the original (non-permuted) Random Forest model, and #(*) indicates the number of the element of *D^perm^* satisfying the inequality. Similar formulas were used to calculate the *p*-values associated with the other measures (accuracy, sensitivity, and specificity).

Variable importance was established suing the Mean Decrease Gini index as customary for Random Forest modeling [125]; it is a measure of how a variable contributes to the homogeneity of the nodes and leaves in the model: the higher the value of the index, the higher the importance of the variable in the model. Statistical significance of the variable importance (*p*-value) was obtained using a permutation approach as implemented in the ‘rfPermute’ R package. 

#### 4.7.5. Network Analysis

##### Inference of Association Networks

The networks of association among lipoproteins and lipid main fractions were built using the Probabilistic Context Likelihood of Relatedness on Correlations (PCLRC) algorithm [25] used in combination with a Gaussian Graphical Model (GMM) to replace pairwise correlations between lipids with partial correlations. In PCLRC, resampling is used to estimate robust correlations based on the Context Likelihood of Relatedness approach [126], which estimates the relevance of the associations between two lipids by considering background associations. The algorithm returns an *m* × *m* probability matrix **P**, containing the likelihood 0 ≤ *p_ij_* ≤ 1 of each observed association (i.e., partial correlation) *r_ij_* between the *m* variables (lipid fractions and lipoproteins). Significant associations are defined as:

(2) $$r_{ij}=\left\{\begin{array}{c} r_{ij}\;\;\;\;if\;p_{ij}\;\geq 0.95\\ 0\;\;\;\;if\;p_{ij}<0.95 \end{array}\right..$$

The algorithm was used with its default parameters (type of correlation *corr.type* = Pearson; number of resampling iterations *Niter* = 1000; the fraction of samples to be considered at each iteration *frac* = 0.75 and fraction of the total prediction interactions to be kept at each iteration *rank.thr* = 0.3). All networks are undirected and represented as an *m* × *m* adjacency matrix **M**, populated by interactions (edges) between lipid *i* and *j* (nodes).

##### Gaussian Graphical Modeling

Partial correlations were estimated using a Gaussian Graphical Model with the GeneNet approach [37] as implemented in the GeneNet R package [37,127]. GeneNet allows estimation of a GMM from a small sample of high-dimensional data in a computationally and statistically efficient way. It uses an analytic shrinkage estimation of covariance and partial correlation matrices and performs optimal model selection based on local false discovery rate multiple testing. The edges (i.e., the associations) between nodes (i.e., lipoproteins and lipid main fractions) to be included in the final association network are selected using a computational algorithm depending on the relative values of the pairwise partial correlations. For more details about the GeneNet algorithm implementation, we refer to the original publication [37]. 

##### Differential Network Analysis

The connectivity of each node (lipid) *i* in a given association network is defined as:

(3) $${Χ}_{i}=\left(\overset{J}{\underset{j=1}{\sum }}\left|r_{ij}\right|\right)-1.$$

The differential connectivity Δ*_i_* for lipid *i* is obtained by subtracting the connectivity of one network from the other, for example:

(4) $$\Delta_{i}^{men,\;women}=\left|{Χ}_{i}^{men}-{Χ}_{i}^{women}\right|$$

The differential connectivity values Δ_i_ were transformed to *z*-scores:

(5) $$z\left(\Delta_{i}\right)=\left(\Delta_{i}-\frac{\sum_{i}\Delta_{i}}{m}\right)/\sigma$$

where *m* is the number of lipoprotein and lipid fractions (*m* = 21) and is the standard deviation calculated over the Δ_i_ values. We considered differentially connected those lipoprotein and lipid fractions with *z*(Δ_i_) > 2.

##### Network Topology Measures

The topology of the networks was analyzed based on several measurements besides the connectivity. The used measurements are: *Average Shortest Path Length, Betweenness Centrality*, *Closeness Centrality*, *Clustering Coefficient*, *Degree*, *Eccentricity*, *Neighborhood Connectivity*, *Number of Directed Edges*, *Radiality*, *Stress*, *Topological Coefficient*. All topological measures are defined in Appendix A**.**

#### 4.7.6. Covariance Simultaneous Component Analysis

The adjacency matrices created by the PCLRC are compared using Covariance Simultaneous Component Analysis (COVSCA) [128], which is a component model to analyze simultaneously communalities and differences across a set of S_k_ (*k* = 1, 2, …, *K*), which are approximated as a linear combination of *L* ≪ *K* low-dimensional prototypes in the form:

(6) $$S_{k}=\overset{L}{\underset{l=1}{\sum }}c_{kl}Z_{l}Z_{l}^{T}$$

where *c_kl_* ≥ 0 (*l* = 1, 2, …, *L*) are weight coefficients, and **Z**_l_**Z**_l_^T^ are the prototypical symmetric matrices that consist of loading **Z** of size J × R_l_ that hold simultaneously for all S*_k_*. We fit a model with two rank-2 prototype matrices as the best compromise between the goodness-of-fit (%) and complexity of the model (100% for perfect fit and 0 for total lack of fit) and the model’s complexity (rank of the prototype matrices). 

Loadings were transformed to *z*-scores (see Method Section 4 *Differential network analysis* for details). Only loadings with *z* ≥ 1 were retained in the analysis.

#### 4.7.7. Software

Calculations were performed in R [129] and Matlab 2019b (The MathWorks, Natick, MA, USA, 2017). The function ‘prcomp’ from the R package ‘stats’ (version 4.0.1.) was used for the Principal Components Analysis. The function ‘wilcox.test’ from the R package ‘’stats’’ (version 4.0.1) was used for the Wilcoxon rank-sum test. The function ‘p.adjust’ from the package ‘stats’ native to R [129] was used for correcting the *p*-values for multiple testing. The function ‘randomForest’ from the package ‘randomForest’ [130] was used for the Random Forest. The ‘rfPermute’ function from the ‘rfPermute’ package [131] was used to obtain *p*-values for the Mean Decrease Gini indexes.

ROC analysis was performed using the R package pROC [132].

The function ‘ggm.estimate.pcor’ from the package ‘GeneNet’ [127] (version 1.2.15) was used for estimation of the partial correlations. For the Random Forest models, the accuracy, sensitivity, and specificity were calculated using the ‘confusionMatrix’ function from the ‘caret’ R package [133]. The AUC was calculated using the ‘colAUC’ function from the ‘caTools’ R package. 

Code for network inference using GMM, differential network analysis and Covariance Simultaneous Component Analysis is available at systemsbiology.nl under the software tab.

Cytoscape [134] (version 3.7.0) was used for network visualization; the Network Analyzer [135] Cytoscape plugin [135] was used to calculate the topological measures.

## 5. Conclusions

In this study, we have identified relevant alterations in the levels and in the pattern of association of lipoprotein fractions in relation to sex and age, using univariate and multivariate statistics and differential network analysis. These observations from the correlation networks, in turn, point to the underlying structure of metabolic mechanisms, indicating a possible age-dependent remodulation of lipase lipoprotein activity in men and a change in the mechanisms controlling the ratio between esterified and non-esterified cholesterol (through the activity of LCAT) in both men and women. The present article highlights the effectiveness of plasma lipidomics by NMR as a powerful (but still underrated with respect to, e.g., metabolomics) discovery tool to explore individuals’ characteristics at the biochemical level. As such, it could rapidly evolve into new clinical tools for clinical research and personalized medicine approaches. It is plausible that, in the near future, plasma lipidomics will expand the common clinical paradigm (which usually includes only TC, HDL, and LDL) to include a more comprehensive lipidomic profile to provide a more accurate characterization of patients’ pathophysiological conditions.

## Figures and Tables

**Table 1:** Age characteristics of the healthy blood donor volunteers in this study. Women (W) and men (M) were stratified into young and old women (YW, OW) and young and old men (YM, OM) by taking the lower 33% and upper 67% percentiles of the age distribution among women and men (cfr. with Table 1 and Figure 1 from [17]). The term “old” is used here as a placeholder given the relatively young age of the study population.

Group Name	Age Group	Subjects (*n*)	Median Age (Years)
W	Women (all)	183	43
YW	Young women (<37 yrs)	56	27
OW	Old women (>48 yrs)	60	55
M	Men (all)	661	40
YM	Young men (<35 yrs)	216	29
OM	Old men (>45 yrs)	213	52

**Table 2:** Comparison of the concentrations of lipoproteins and lipid main fractions between men and women, young men and old men, and young women and old women (Wilcoxon rank-sum test). * indicates statistically significant differences at α = 0.05 level after Bonferroni correction for multiple testing (actual α = 0.05/21 = 0.0024). Age groups are defined in Table 1. Concentrations are in mg/dL.

		Concentration					
	Lipid/Lipoprotein	Men	Women	Young Men	Old Men	Young Women	Old Women
1	Apo-A1, HDL	156.6 *	176.8 *	155.3	160.1	170.3	184.0
2	Apo-A2, HDL	26.8 *	30.3 *	26.2	28.1	28.6	32.2
3	Apo-B, IDL	3.1 *	2.6 *	2.6 *	3.9 *	1.8 *	3.2 *
4	Apo-B, LDL	61.1	63.2	55.9 *	67.8 *	51.9 *	74.1 *
5	Apo-B, VLDL	6.6 *	5.0 *	5.4 *	8.1 *	4.7	5.2
6	Cholesterol, HDL	66.7 *	76.9 *	66.4	67.7	73.8	80.1
7	Cholesterol, IDL	6.9 *	4.7 *	5.6 *	8.9 *	3.1	6.0
8	Cholesterol, LDL	154.8	146.1	153.3	159.3	124.6 *	165.7 *
9	Cholesterol, VLDL	14.0 *	9.0 *	10.6 *	18.1 *	8.4	8.9
10	Free Cholesterol, HDL	13.4 *	16.8 *	12.9	13.8	16.2	17.5
11	Free Cholesterol, IDL	1.9 *	1.4 *	1.6 *	2.6 *	0.90	1.8
12	Free Cholesterol, LDL	40.0	38.8	38.8	41.6	33.4 *	44.2 *
13	Free Cholesterol, VLDL	7.7 *	5.7 *	5.8 *	9.7 *	5.5	5.9
14	Phospholipids, HDL	74.7 *	93.3 *	73.2	77.0	91.0	96.3
15	Phospholipids, IDL	5.2 *	3.9 *	4.5 *	6.435 *	2.9	4.7
16	Phospholipids, LDL	77.7	76.3	75.6	81.1	66.1 *	86.0 *
17	Phospholipids, VLDL	19.6 *	14.0 *	15.7 *	24.1 *	13.6	14.2
18	Triglycerides, HDL	7.3 *	9.2 *	6.4 *	8.2 *	9.5	9.1
19	Triglycerides, IDL	7.5	5.2	4.8 *	10.7 *	4.4	5.5
20	Triglycerides, LDL	5.49 *	8.1 *	4.0 *	7.5 *	6.2	9.5
21	Triglycerides, VLDL	45.4 *	29.7 *	31.5 *	61.1 *	27.1	29.8

**Table 3:** ROC analysis of the concentrations of lipoproteins and lipid main fractions in men and women. Age groups are defined in Table 1. Concentrations are in mg/dL. Adj stands for adjusted. CI indicates the 95% Confidence Interval.

	Lipid/Lipoprotein	AUC	CI AUC Lower	CI AUC Upper	Threshold	Accuracy	Specificity	Sensitivity	*p*-Value	Adjusted *p*-Value
1	Apo-A1, HDL	0.689	0.644	0.735	166.3	0.674	0.681	0.650	<0.001	<0.001
2	Apo-A2, HDL	0.656	0.612	0.701	28.6	0.633	0.634	0.628	<0.001	<0.001
3	Apo-B, IDL	0.605	0.557	0.652	1.8	0.684	0.755	0.426	<0.001	0.001
4	Apo-B, LDL	0.524	0.477	0.572	65.4	0.582	0.622	0.437	0.315	1.000
5	Apo-B, VLDL	0.640	0.595	0.685	4.6	0.642	0.670	0.541	<0.001	<0.001
6	Cholesterol, HDL	0.715	0.672	0.758	76.6	0.755	0.820	0.519	<0.001	<0.001
7	Cholesterol, IDL	0.644	0.599	0.690	5.3	0.600	0.585	0.650	<0.001	<0.001
8	Cholesterol, LDL	0.554	0.507	0.601	153.6	0.524	0.501	0.607	0.026	1.000
9	Cholesterol, VLDL	0.648	0.605	0.692	12.4	0.533	0.469	0.765	<0.001	<0.001
10	Free Cholesterol, HDL	0.784	0.747	0.820	15.5	0.763	0.793	0.656	<0.001	<0.001
11	Free Cholesterol, IDL	0.637	0.591	0.683	1.5	0.597	0.585	0.639	<0.001	<0.001
12	Free Cholesterol, LDL	0.539	0.490	0.588	35.7	0.620	0.676	0.415	0.108	1.000
13	Free Cholesterol, VLDL	0.628	0.585	0.671	7.7	0.514	0.439	0.787	<0.001	<0.001
14	Phospholipids, HDL	0.793	0.756	0.830	85.1	0.763	0.785	0.683	<0.001	<0.001
15	Phospholipids, IDL	0.616	0.571	0.661	4.2	0.592	0.581	0.634	<0.001	<0.001
16	Phospholipids, LDL	0.524	0.476	0.571	73.1	0.552	0.569	0.492	0.325	1.000
17	Phospholipids, VLDL	0.666	0.623	0.709	16.8	0.604	0.576	0.705	<0.001	<0.001
18	Triglycerides, HDL	0.623	0.577	0.669	6.8	0.532	0.486	0.699	<0.001	<0.001
19	Triglycerides, IDL	0.578	0.535	0.622	7.7	0.444	0.342	0.814	0.001	0.071
20	Triglycerides, LDL	0.622	0.577	0.667	5.1	0.582	0.570	0.623	<0.001	<0.001
21	Triglycerides, VLDL	0.627	0.584	0.670	42.8	0.527	0.449	0.809	<0.001	<0.001

**Table 4:** ROC analysis of the concentrations of lipoproteins and lipid main fractions in young and old men. Age groups are defined in Table 1. Concentrations are in mg/dL. Adj stands for adjusted. CI indicates the 95% Confidence Interval.

	Lipid/Lipoprotein	AUC	CI AUC Lower	CI AUC Upper	Threshold	Accuracy	Specificity	Sensitivity	*p*-Value	Adjusted *p*-Value
1	Apo-A1, HDL	0.553	0.498	0.607	178.4	0.562	0.230	0.889	0.059	1.000
2	Apo-A2, HDL	0.581	0.527	0.635	27.3	0.578	0.549	0.606	0.004	0.227
3	Apo-B, IDL	0.710	0.660	0.759	2.8	0.681	0.765	0.597	<0.001	<0.001
4	Apo-B, LDL	0.665	0.614	0.716	65.1	0.636	0.563	0.708	<0.001	<0.001
5	Apo-B, VLDL	0.720	0.672	0.768	6.9	0.671	0.577	0.764	<0.001	<0.001
6	Cholesterol, HDL	0.530	0.475	0.585	62.7	0.534	0.653	0.417	0.281	1.000
7	Cholesterol, IDL	0.688	0.638	0.739	7.7	0.650	0.559	0.741	<0.001	<0.001
8	Cholesterol, LDL	0.546	0.491	0.601	172.1	0.562	0.437	0.685	0.100	1.000
9	Cholesterol, VLDL	0.686	0.636	0.736	16.2	0.650	0.502	0.796	<0.001	<0.001
10	Free Cholesterol, HDL	0.586	0.533	0.640	11.4	0.573	0.789	0.361	0.002	0.126
11	Free Cholesterol, IDL	0.684	0.633	0.734	2.4	0.646	0.498	0.792	<0.001	<0.001
12	Free Cholesterol, LDL	0.591	0.537	0.645	41.7	0.592	0.507	0.676	0.001	0.069
13	Free Cholesterol, VLDL	0.733	0.686	0.780	7.3	0.695	0.676	0.713	<0.001	<0.001
14	Phospholipids, HDL	0.575	0.521	0.629	74.9	0.566	0.535	0.597	0.007	0.438
15	Phospholipids, IDL	0.659	0.607	0.710	5.3	0.634	0.587	0.681	<0.001	<0.001
16	Phospholipids, LDL	0.591	0.537	0.644	79.6	0.590	0.563	0.616	0.001	0.074
17	Phospholipids, VLDL	0.719	0.671	0.768	20.0	0.688	0.638	0.736	<0.001	<0.001
18	Triglycerides, HDL	0.674	0.623	0.724	7.2	0.639	0.620	0.657	<0.001	<0.001
19	Triglycerides, IDL	0.720	0.672	0.768	2.7	0.678	0.854	0.505	<0.001	<0.001
20	Triglycerides, LDL	0.683	0.633	0.734	6.4	0.662	0.563	0.759	<0.001	<0.001
21	Triglycerides, VLDL	0.713	0.664	0.761	43.1	0.676	0.634	0.718	<0.001	<0.001

**Table 5:** ROC analysis of the concentrations of lipoprotein main fractions in young and old women. Age groups are defined in Table 1. Concentrations are in mg/dL. Adj stands for adjusted. CI indicates the 95% Confidence Interval.

	Lipid/Lipoprotein	AUC	CI AUC Lower	CI AUC Upper	Threshold	Accuracy	Specificity	Sensitivity	*p*-Value	Adjusted *p*-Value
1	Apo-A1, HDL	0.621	0.516	0.727	171.6	0.655	0.733	0.571	0.024	1.000
2	Apo-A2, HDL	0.665	0.565	0.764	25.4	0.647	0.950	0.321	0.002	0.143
3	Apo-B, IDL	0.704	0.608	0.799	1.7	0.672	0.750	0.589	<0.001	0.010
4	Apo-B, LDL	0.793	0.710	0.875	55.5	0.750	0.867	0.625	<0.001	<0.001
5	Apo-B, VLDL	0.560	0.454	0.666	4.1	0.586	0.633	0.536	0.264	1.000
6	Cholesterol, HDL	0.608	0.505	0.711	70.1	0.621	0.800	0.429	0.045	1.000
7	Cholesterol, IDL	0.682	0.585	0.779	3.5	0.690	0.700	0.679	0.001	0.042
8	Cholesterol, LDL	0.755	0.667	0.844	127.5	0.716	0.850	0.571	<0.001	<0.001
9	Cholesterol, VLDL	0.473	0.366	0.579	1.3	0.517	0.150	0.911	0.613	1.000
10	Free Cholesterol, HDL	0.603	0.499	0.707	13.6	0.612	0.950	0.250	0.056	1.000
11	Free Cholesterol, IDL	0.683	0.586	0.780	1.0	0.681	0.700	0.661	0.001	0.040
12	Free Cholesterol, LDL	0.802	0.721	0.883	34.4	0.767	0.900	0.625	<0.001	<0.001
13	Free Cholesterol, VLDL	0.544	0.438	0.650	3.7	0.560	0.733	0.375	0.414	1.000
14	Phospholipids, HDL	0.560	0.453	0.667	78.4	0.612	0.883	0.321	0.269	1.000
15	Phospholipids, IDL	0.681	0.584	0.779	4.7	0.664	0.517	0.821	0.001	0.049
16	Phospholipids, LDL	0.782	0.698	0.866	77.1	0.733	0.683	0.786	<0.001	<0.001
17	Phospholipids, VLDL	0.538	0.432	0.645	8.6	0.578	0.767	0.375	0.478	1.000
18	Triglycerides, HDL	0.506	0.397	0.615	6.7	0.569	0.817	0.304	0.916	1.000
19	Triglycerides, IDL	0.634	0.530	0.737	1.9	0.672	0.867	0.464	0.013	0.820
20	Triglycerides, LDL	0.674	0.575	0.772	3.7	0.664	0.867	0.446	0.001	0.079
21	Triglycerides, VLDL	0.566	0.460	0.671	18.9	0.586	0.650	0.518	0.223	1.000

**Table 6:** Quality measures for the Random Forest classification models used to discriminate between the blood lipoprotein main fraction profiles of the study groups. Statistical significance was assessed using a permutation test.

Random Forest Model	Accuracy (*p*-Value)	Specificity (*p*-Value)	Sensitivity (*p*-Value)	AUC (*p*-Value)
Women vs men	0.776 (0.001)	0.761 (0.001)	0.780 (0.001)	0.826 (0.001)
Young vs old men	0.746 (0.001)	0.741 (0.001)	0.751 (0.001)	0.810 (0.001)
Young vs old women	0.716 (0.001)	0.679 (0.002)	0.750 (0.001)	0.762 (0.005)

**Table 7:** Summary overview of the results of the integrated analysis performed: U, univariate analysis of lipid and lipoprotein concentration using Mann–Whitney–Wilcoxon test (see Table 2); RF, Random Forest predictive modeling (see Table 6 and Figure 3); Differential connectivity analysis (see Figure 7); C, Covariance simultaneous component analysis (see Figure 5); T, concordance among the analysis. * indicates a statistically significant or relevant result.

		Men vs Women					Young Men vs Old Men					Young Women vs Old Women				
	Lipid/Lipoprotein	U	RF	D	C	T	U	RF	D	C	T	U	RF	D	C	T
1	Apo-A1 HDL	*	*	*	*	4				*	1				*	1
2	Apo-A2 HDL	*		*		2					0			*		1
3	Apo-B IDL	*	*			2	*	*			2	*				1
4	Apo-B LDL					0	*		*	*	3	*	*		*	3
5	Apo-B VLDL	*				1	*		*		2					0
6	Cholesterol HDL	*	*			2				*	1				*	1
7	Cholesterol IDL	*	*			2	*				1					0
8	Cholesterol LDL					0					0	*				1
9	Cholesterol VLDL	*				1	*	*			2					0
10	Free cholesterol HDL	*	*	*		3			*	*	2				*	1
11	Free cholesterol IDL	*	*	*	*	4	*				1					0
12	Free cholesterol LDL			*		1					0	*	*			2
13	Free cholesterol VLDL	*			*	2	*	*			2					0
14	Phospholipids HDL	*	*			2				*	1				*	1
15	Phospholipids IDL	*	*			2	*				1					0
16	Phospholipids LDL					0				*	1	*	*		*	3
17	Phospholipids VLDL	*			*	2	*	*		*	3				*	1
18	Triglycerides HDL	*	*			2	*				1					0
19	Triglycerides IDL					0	*		*		2					0
20	Triglycerides LDL	*	*			2	*	*			2					0
21	Triglycerides VLDL	*	*			2	*				1					0

## Data Availability

Lipoprotein and lipid fraction data are available at the NIH Common Fund’s National Metabolomics Data Repository (NMDR) website, the Metabolomics Workbench, https://www.metabolomicsworkbench.org, where it has been assigned Project ID ST001785. The data can be accessed directly via its Project https://doi.org/ST001785.

## Appendix A

The *average shortest path length* of a node n is:

(A1) $$ASPL_{n}=\frac{\sum_{1}^{p}L\left(n,m\right)}{p},\;$$

where $k_{n}$ is the degree of node n, $L\left(n,m\right)$ is the length of the shortest path between n and any other node m, and p is the number of nodes excluding n [136].

The *betweenness centrality* of a node n is:

(A2) $$C_{b}\left(n\right)=\sum_{s\neq n\neq t}\left(\frac{\sigma_{st}\left(n\right)}{\sigma_{st}}\right),\;$$

where s and t are nodes in the network different from n, $\sigma_{st}$ denotes the number of shortest paths from s to t, and $\sigma_{st}\left(n\right)$ is the number of shortest paths from s to t that n lies on [137].

The *closeness centrality* of a node n is:

(A3) $$C_{c}\left(n\right)=\frac{1}{ASPL_{n}},\;$$

where $ASPL_{n}$ is the average shortest path length of node n. The closeness centrality is a number between 0 and 1 [137]

The *clustering coefficient* of a node n in undirected networks is:

(A4) $$C_{n}=\frac{2e_{n}}{k_{n}\left(k_{n}-1\right)},\;$$

where $k_{n}$ is the number of neighbours of n, and $e_{n}$ is the number of connected pairs between all neighbors of n [138].

The *degree* of a node n is:

(A5) $$k_{n},$$

where $k_{n}$ is the number of edges linked to n [139].

The *eccentricity* of a node n is:

(A6) $$\underset{0\leq x<\infty }{\max}\left(L\left(n,m\right)\right),$$

where $L\left(n,m\right)$ is the length of the shortest path between n and any other node m [140].

The *neighborhood connectivity* of a node n is:

(A7) $$\frac{\sum_{1}^{k_{n}}k_{b}}{k_{n}},\;$$

where $k_{n}$ is the degree of node n, and b is each neighbor of n [141].

The *radiality* of a node n is:

(A8) $$C_{R}\left(v\right)=\frac{\sum_{t\in V}\left(D\left(G\right)+1-d_{G}\left(v,t\right)\right)}{\left(n-1\right)\cdot D\left(G\right)},\;$$

where $D\left(G\right)=max_{st\in V}d_{G}\left(s,t\right)$.

The *stress centrality* of a node n is:

(A9) $$C_{s}\left(n\right)=\underset{s\neq n\neq t}{\sum }\sigma_{st}\left(n\right),\;$$

where s and t are nodes in the network different from n, and $\sigma_{st}\left(n\right)$ is the number of shortest paths from s to t that n lies on [142].

The *topological coefficient* of a node n is:

(A10) $$TC_{n}=avg\left(\frac{J\left(n,m\right)}{k_{n}}\right),\;$$

where $J\left(n,m\right)$ denotes the number of nodes to which both n and m are linked, and $k_{n}$ is the number of links of node *n* [143].
